# Prediction of the 10-Year Risk of Cardiovascular Diseases Among Patients in Primary Health Care Centers in Eastern Province, Saudi Arabia

**DOI:** 10.7759/cureus.47551

**Published:** 2023-10-23

**Authors:** Mohammed Almulhim, Jumanah Alqattan, Adnan Almajed, Mohammed A Alkhars, Abdullah A Alhafith, Mohammed S Alajmi, Razan Alhussain, Sayed Ali, Eman Elsheikh, Muthana Al Sahlawi

**Affiliations:** 1 Internal Medicine, College of Medicine, King Faisal University, Al-Ahsa, SAU; 2 College of Medicine, King Faisal University, Al-Ahsa, SAU; 3 Pediatrics, Maternity and Children Hospital, Dammam, SAU; 4 Family and Community Medicine, King Faisal University, Al-Ahsa, SAU; 5 Cardiology, College of Medicine, Tanta University, Tanta, EGY; 6 Internal Medicine, King Faisal University, Al-Ahsa, SAU

**Keywords:** kingdom of saudi arabia (ksa), 10-year cardiovascular risk, risk prediction, framingham risk score (frs), cardiovascular disease (cvd)

## Abstract

Background

Cardiovascular diseases (CVDs), primarily coronary artery disease (CAD) and stroke, stand as a leading cause of morbidity and mortality globally. Our objective was to predict the 10-year risk of CVD in the Eastern Province of Saudi Arabia.

Methods

This cross-sectional study was conducted in eight randomly selected primary healthcare centers using cluster sampling based on geographical location in Saudi Arabia's Eastern Province, specifically the Al-Ahsa region. The study aimed to assess the risk of developing CVD in the next 10 years among patients with at least one cardiovascular risk factor. Patients visiting the healthcare centers for checkups filled out the Framingham Cardiovascular Disease (10-year risk) score questionnaire.

Results

Of the 665 patients enrolled, 54.4% were female. The average age of the patients was 54.2 (SD 8.48) years. The overall average Framingham Risk Score (FRS) percentage was 19.2% (SD 15.4%). In terms of 10-year CVD risk, 34.6% of the patients were at high risk, 31.6% were at moderate risk, and 33.8% were considered low-risk individuals. Factors associated with a higher risk of CVD included older age, male gender, lower educational attainment, smoking, normal BMI, stage 2 hypertension, and diagnoses of hypertension, diabetes, and obesity.

Conclusion

Utilizing the FRS, it was determined that older men with lower educational levels had a higher 10-year risk of developing CVD. Furthermore, CVD risk factors such as diabetes, hypertension, obesity, and smoking were associated with individuals' CVD risk. Considering the ease of use and applicability of the FRS in daily clinical practice, as well as its potential to identify high-risk individuals, a more systematic implementation in general practice appears to be warranted.

## Introduction

Coronary artery disease (CAD) is the most common cardiovascular disease (CVD) and the leading global cause of mortality [1]. In recent years, CVD morbidity and mortality have increased greatly, from 271 million to 523 million and from 12.1 million to 18.6 million annually, respectively [2]. In Saudi Arabia, CVD has the highest mortality rate among all diseases, accounting for 37% of total deaths in 2018 [3]. The main risk factors leading to CVD are hypertension, diabetes, dyslipidemia, obesity, smoking, and a sedentary lifestyle. All of these are very common in the area, which is the main cause of the high incidence of the disease [4]. Early detection and modification of these risk factors will help reduce the burden of CVD and will lead to a better quality of life for the patients [5].

There are various risk-assessment tools available that can fairly and accurately examine the effects of numerous CV risk factors and generate an estimate of CV risk, which can subsequently be used to design disease management interventions and disease prevention programs for those who are at high risk of CVD [6,7]. The Framingham Risk Score (FRS) is the most widely utilized of these tools [6,7]. It evaluates the chance or likelihood of developing CVD in individuals [8]. Predictors included in the Framingham model were age, gender, systolic blood pressure (SBP), high-density lipoprotein cholesterol (HDL-C), total cholesterol (TC), smoking status, and history of diabetes [9]. The FRS was developed as a validated method in North America to estimate the probability of CVD in asymptomatic patients [10]. A percentage can be calculated using the FRS to determine a 10-year risk score, which can subsequently be used to help determine whether or not to start lipid-lowering treatment for primary prevention. If the FRS is less than 10%, it is classified as low risk; moderate risk if it falls between 10% and 19%, and high risk if it is 20% or above [11]. This risk score not only indicates the potential benefits of prevention but also aids patients and healthcare professionals in determining the necessity of lifestyle modifications or preventive medical interventions. It can also help educate patients by showing which men and women are at a higher risk for future cardiovascular events [12].

To reduce mortality from CVD, it is essential to encourage high-risk patients to take statins. Statins are especially beneficial for those with high-risk scores [13]. It has been demonstrated that treating people with high-risk scores, but normal cholesterol levels can reduce mortality [14,15].

To evaluate the challenges that might face the healthcare system in the next 10 years and work to improve the outcomes, this study aims to predict the 10-year risk of CVD using the FRS among individuals with at least one risk factor in a primary healthcare setting in Al-Ahsa, Eastern Province, Saudi Arabia.

## Materials and methods

Study design and setting

A cross-sectional study was conducted in Al-Ahsa, the largest city in the Eastern Province of Saudi Arabia, in eight primary healthcare centers (PHCs) from August to December 2022. The PHCs were chosen based on their geographical location. Two primary healthcare centers of the north, east, center, and south were chosen to reliably represent the region.

Study population and data collection

Our research assessed the likelihood of developing CVD over the next 10 years in individuals aged 30 to 74 years with no history of chronic heart disease but with one or more cardiovascular risk factors, including hypertension, diabetes, obesity, and smoking. Data from patients visiting the eight PHCs during the study were retrieved from the PHC's database system. The data were verified by the participants, and informed consent was obtained through an interview session to ensure data accuracy. The information collected included age, sex, education level, weight, height, SBP at the time of the appointment, smoking status, presence of diabetes, and treatment status for hypertension. Out of the 900 patients visiting the PHCs, 665 patients met the criteria for this study.

Based on the acquired data, we utilized the non-laboratory version of Framingham's CVD (10-year risk) score calculator to assess the risk of developing CVD in the next 10 years for each participant. Hypertension is diagnosed as stage 1 if the SBP is between 140 and 159 mmHg and/or the diastolic blood pressure is between 90 and 99 mmHg, and as stage 2 if the SBP is 160 mmHg or higher and/or the diastolic blood pressure is 100 mmHg or higher [16]. For diabetes, the diagnosis can be made with a fasting plasma glucose level of 126 mg/dL or greater, an A1C level of 6.5% or greater, a random plasma glucose level of 200 mg/dL or greater, or a 75-g two-hour oral glucose tolerance test with a plasma glucose level of 200 mg/dL or greater. Results should be confirmed with repeated testing on a subsequent day. However, a single random plasma glucose level of 200 mg/dL or greater with typical signs and symptoms of hyperglycemia is diagnostic [17].

BMI was calculated to determine obesity, with categories including underweight, normal weight, overweight, obese (class I), and morbidly obese (classes II and III): <18.5, 18.5-24.9, 25-29.9, 30-34.9, and 35 kg/m^2^, respectively [18]. The FRS calculator consists of seven categories: sex, age, SBP at the time of the appointment, treatment for hypertension, smoking status, diabetes, and body mass index [11].

Statistical analysis

Descriptive statistics were presented as numbers and percentages for all qualitative variables, while mean and standard deviation were used to present all quantitative variables. The comparison between the FRS and the patient’s demographic characteristics was conducted using the Mann-Whitney Z-test and Kruskal-Wallis H-test. The normality of the data was assessed through the Shapiro-Wilk test and Kolmogorov-Smirnov test. The FRS (%) follows the non-normal distribution. Thus, there were nonparametric tests used. In order to demonstrate statistical significance, a P-value of 0.05 was used. IBM SPSS Statistics for Windows, Version 26 (Released 2019; IBM Corp., Armonk, New York) was used to examine each and every statistical data set.

## Results

Data from 665 patients who visited PHCs in Al-Ahsa and met the inclusion criteria were analyzed in this study. The mean age of the patients was 54.2 years (SD 8.48), with 40.8% aged between 51 and 60 years. More than half were females (54.4%), and 33.7% visited PHC centers in Al-Ahsa South. 60.6% of the patients held university degrees. Patients classified as obese constituted 56.4% of the sample. The prevalence of smoking among patients was 10.2%. Regarding SBP levels, 46% were categorized as having stage 2 hypertension (mean value: 139.6; SD 20.2). The most associated risk factor was obesity (55.6%), followed by hypertension (26.5%).

**Figure 1:**
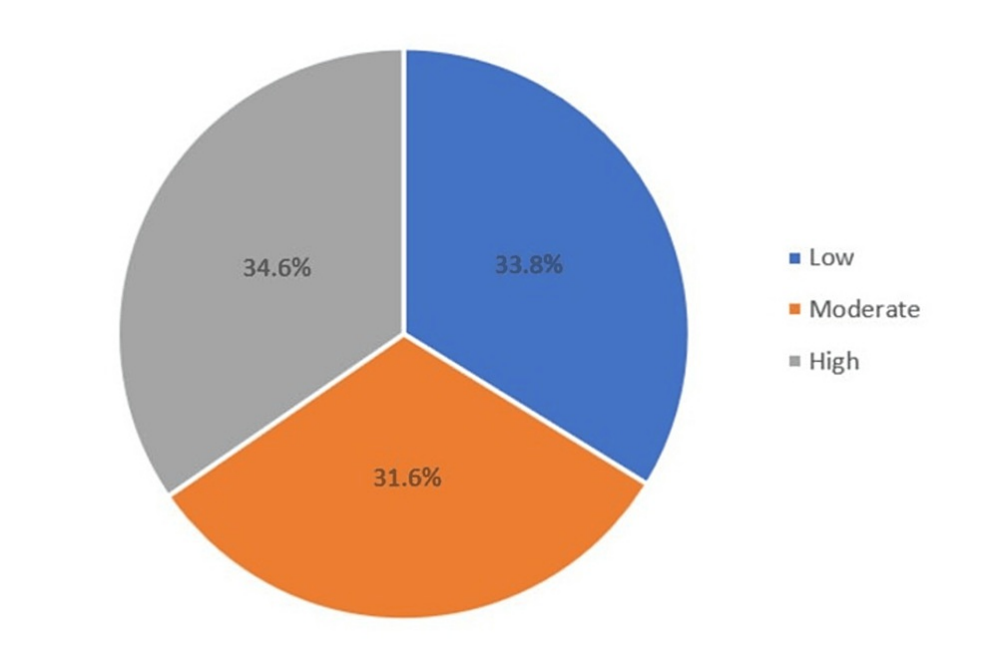
Levels of FRS: low risk, moderate risk, and high risk.

In the assessment of CVD risk according to the Framingham 10-year CVD risk score, 34.6% (n=230) were categorized as high risk, 31.6% as moderate risk (n=210), and 33.8% as low-risk (n=225) (Table 1, Figure 1). When measuring the differences in the percentage of Framingham score in relation to patient demographic characteristics (Table 2), it was found that an increase in the percentage of FRS was more associated with the older age group (Z=13.579; P<0.001), male gender (Z=8.148; P<0.001), those who visited Al-Ahsa North (H=10.598; P=0.014), being married (Z=2.064; P=0.039), having lower educational attainment (H=25.551; P<0.001), having a normal body mass index (H=7851; P=0.005), being a smoker (Z=4.387; P<0.001), having stage 2 hypertension (H=208.944; P<0.001), and those who were diagnosed with hypertension (Z=12.396; P<0.001), diabetes (Z=11.336; P<0.001), and obesity (Z=2.087; P=0.037), while those with no diagnosis were less likely to have an increased percentage of FRS (Z=8.013; P<0.001).

**Table 1:** Patients’ demographic characteristics. ^†^Some patients have multiple diagnoses. PHC, primary healthcare center; SBP, systolic blood pressure; FRS, Framingham Risk Score.

Study variables	N (%)
Age group (mean ± SD)	54.2 ± 8.48
35–50 years	224 (36.7%)
51–60 years	271 (40.8%)
>60 years	150 (22.6%)
Gender	
Male	303 (45.6%)
Female	362 (54.4%)
PHC location in Al-Ahsa	
South	224 (33.7%)
North	210 (31.6%)
Center	156 (23.5%)
East	75 (11.3%)
Marital status	
Single	105 (15.8%)
Married	560 (84.2%)
Educational level	
No formal education	136 (20.5%)
University	403 (60.6%)
Postgraduate	126 (18.9%)
BMI level (mean ± SD)	31.7 ± 5.86
Normal (18.5–24.9 kg/m^2^)	43 (06.5%)
Overweight (25–29.9 kg/m^2^)	247 (37.1%)
Obese (≥30 kg/m^2^)	375 (56.4%)
Smoking	
Yes	68 (10.2%)
No	597 (89.8%)
SBP level	139.6 ± 20.2
Normal (<120 mmHg)	100 (15.0%)
Elevated (120–129 mmHg)	104 (15.6%)
Stage 1 hypertension (130–139 mmHg)	155 (23.3%)
Stage 2 hypertension (≥140 mmHg)	306 (46.0%)
Diagnosis^†^	
Hypertension	176 (26.5%)
Diabetes	137 (20.6%)
Obesity	370 (55.6%)
None	193 (29.0%)
FRS level	19.2 ± 15.4
Low risk (<10%)	225 (33.8%)
Moderate risk (10–20%)	210 (31.6%)
High risk (>20%)	230 (34.6%)

**Table 2:** Association between FRS% and patients’ demographic characteristics. ^†^Some patients have multiple diagnoses. ^a^P-value has been calculated using the Mann-Whitney Z-test. ^b^P-value has been calculated using the Kruskal-Wallis H-test. **Significant at P < 0.05. PHC, primary healthcare center; SBP, systolic blood pressure; FRS, Framingham Risk Score.

Factors	FRS%, mean ± SD	Z/H-test	P-value
Age group			
<55 years	12.1 ± 9.04	Z=13.579	<0.001**^,^^a^
≥55 years	26.8 ± 16.9		
Gender			
Male	23.9 ± 16.5	Z=8.148	<0.001**^,a^
Female	15.4 ± 13.1		
PHC location in Al-Ahsa			
South	18.8 ± 14.7	H=10.598	0.014**^,b^
North	21.0 ± 16.1		
Center	19.2 ± 15.4		
East	15.6 ± 14.7		
Marital status			
Single	16.2 ± 12.7	Z=2.064	0.039**^,a^
Married	19.8 ± 15.8		
Educational level			
No formal education	25.2 ± 19.2	H=25.551	<0.001**^,b^
University	18.5 ± 14.2		
Postgraduate	15.0 ± 12.1		
BMI level			
Normal (18.5–24.9 kg/m^2^)	22.6 ± 15.5	H=7.851	0.005**^,b^
Overweight (25–29.9 kg/m^2^)	17.5 ± 15.5		
Obese (≥30 kg/m^2^)	20.0 ± 15.2		
Smoking			
Yes	26.8 ± 18.3	Z=4.387	<0.001**^,a^
No	18.4 ± 14.8		
SBP Level			
Normal (<120 mmHg)	9.53 ± 9.28	H=208.944	<0.001**^,b^
Elevated (120–129 mmHg)	12.1 ± 9.75		
Stage 1 hypertension (130–139 mmHg)	15.0 ± 15.6		
Stage 2 hypertension (≥140 mmHg)	26.9 ± 16.8		
Diagnosis^†^			
Hypertension	31.8 ± 17.9	Z=12.396	<0.001**^,a^
Diabetes	33.5 ± 18.5	Z=11.336	<0.001**^,a^
Obesity	19.8 ± 15.2	Z=2.087	0.037**^,a^
None	13.0 ± 11.5	Z=8.013	<0.001**^,a^

## Discussion

The purpose of the present study is to determine the 10-year CVD risk of patients visiting PHCs in Al-Ahsa, Saudi Arabia. Based on the Framingham score, the 10-year CVD risk among Al Ahsa patients was high at 34.6%, 31.6% were at moderate risk, and 33.8% were at low risk (mean FRS score: 19.2; SD 15.4). These proportions were expected, given that most participants had co-morbid conditions, and both genders were involved; thus, the percentage of moderate and high-risk participants was high.

Consistent with our findings, Abril-López et al. reported that 59.2% of their population were at high risk for CVD, 29.2% were at intermediate risk, and 15% were at low risk [18]. Another study by Nakhaie et al. found that 95% of office workers were at low risk for CVD, 6.6% at moderate risk, and 2.9% at high 10-year risk of CVD [19]. Among health workers, the 10-year risk of developing CVD was generally low. Based on FRS, 98.3% were considered low risk, 1.0% were moderate risk, and only 0.7% were at high risk [20]. These variations largely differ due to the type of population involved. In our study, patients with at least one CVD risk factor were involved, while in the latter, the study mainly focused on office or health workers. It is expected that participants with underlying diseases have a higher chance of developing CVD in 10 years compared to the normal population.

Our results showed increased CVD risk among older patients, males, patients visiting PHC Al Ahsa North, and those with lower educational levels. In a recent study in Riyadh, Saudi Arabia [21], low activity, prolonged sitting time, and high central obesity were independent significant predictors of intermediate/high CVD risk. Similarly, a higher predicted 10-year CVD risk in males was also reported by Nakhaie et al., and they found a significantly lower 10-year CVD risk among participants with a Master of Science degree or higher and subjects with a normal waist-to-hip ratio [19].

The prevalence of CVD risk factors such as diabetes, high blood pressure readings, and obesity was found among 20.6%, 26.5%, and 55.6%, respectively. Additionally, 10.2% of the patients were smokers, and obese patients accounted for 56.4%. Based on SBP criteria, 46% were classified as having stage 2 hypertension.

These findings align with a study documented by Emamian et al., which revealed that factors associated with CVD risk include diabetes, smoking (only in men), high blood pressure, triglycerides (only in women), TC, waist circumference, and HDL-C [22]. These results are comparable to those of Alhabib et al. [4]. They mentioned that low physical activity, obesity, unhealthy diet, dyslipidemia, hypertension, and diabetes were found in 64.4%, 49.6%, 34.4%, 32.1%, 30.3%, and 25.1% of the population, respectively. Additional contributors included a history of periods of stress (16.9%), feeling sad (15.4%), smoking (12.2%), permanent stress (6.8%), with few cases of coronary heart disease (2.5%), a history of stroke (1%), and a history of heart failure (0.6%), while in a report by Abril-López et al. [23], hypertensive patients who were at risk of CVD had been diagnosed with elevated SBP (47.5%), hypercholesterolemia (39.2%), type 2 diabetes mellitus (15%), and a history of smoking (13.3%).

Limitations

The study's cross-sectional survey base restricts the study's ability to interpret observed relationships causally and to consider risk factors from a dynamic or life-course viewpoint. The nonlaboratory version of the FRS used in this study may misclassify CVD risk when used. However, laboratory blood tests are still challenging, with the measurement of glucose using a capillary blood glucose sensor and the absence of TC and HDL measurements being common in large-scale research conducted in resource-poor nations: The only difference between this score and "laboratory" Framingham's risk factors was that the BMI was utilized in place of the lipid tests (TC and HDL). This approach was initially intended for usage in environments with constrained resources. Pandya et al. demonstrated excellent agreement between the "laboratory" model and the "non-laboratory" Framingham risk categorization [24].

## Conclusions

In conclusion, the study identified a higher risk of developing CVD within a 10-year period using the nonlaboratory FRS among older male patients who were married and had lower educational levels. Additionally, CVD risk factors such as diabetes, hypertension, obesity, and smoking were associated with an increased risk of CVD among the participants. Considering the simplicity and appropriateness of FRS for routine clinical practice, as well as its potential to identify high-risk individuals, greater systematic utilization in general practice appears warranted. We advocate strengthening preventative measures to balance cardiovascular risk factors, such as diabetes and hypertension. Furthermore, Improved CVD mortality reduction depends on the success of efforts to get high-risk people screened using FRS and to take statins.
